# Menstrual Characteristics Associated With Successful Pregnancy Within 6 Months Among Women Planning to Conceive

**DOI:** 10.1097/jnr.0000000000000705

**Published:** 2025-09-30

**Authors:** Tzu Ling CHEN, Li-Yin CHIEN

**Affiliations:** 1 Department of Nurse-Midwifery and Women Health, National Taipei University of Nursing and Health Sciences, Taipei, Taiwan; 2 Institute of Community Health Care, College of Nursing, National Yang Ming Chiao Tung University, Yang-Ming Campus, Taipei, Taiwan

**Keywords:** pre-pregnancy, menstrual cycle, ovulation signs, fertility, time to pregnancy

## Abstract

**Background:**

Enhanced awareness/understanding of menstrual cycle characteristics and the ovulation period can help raise the rate of natural conception among women planning to conceive. The multidimensional nature of menstrual characteristics and their relationship with conception have been rarely addressed in the literature.

**Purpose:**

This study describes five menstrual cycle characteristics and explores their association with conception and time to pregnancy (TTP).

**Methods:**

This prospective longitudinal study was conducted between August 2018 and September 2019, with a total of 289 women participating. All of the participants were planning to become pregnant and were interviewed in person during the baseline examination. Menstrual cycle characteristics were recorded from the time participants joined the study through a minimum of three subsequent cycles. Five cycle characteristics were recorded, including overall length, number of days of bleeding, signs of ovulation, occurrence of dysmenorrhea, and occurrence of blood clots. Signs of ovulation were defined as a drop in basal body temperature lasting 1–2 days accompanied by a positive ovulation test result. Pregnancy outcomes and TTP were recorded over the first 6 months after enrollment as a participant in this study.

**Results:**

Nearly half (44.6%) of the participants became pregnant during the 6-month observation period, and the mean TTP of the sample was 81.9 (*SD*=38.1) days. Based on the 1,321 menstrual cycles recorded in this study, the Cox proportional hazards regression results identified participants aged 30–35 years as more likely to become pregnant than those under 30. Having a menstrual cycle length of 27–32 days, bleeding for 5–6 days, signs of ovulation on Days 13–16, and lack of dysmenorrhea or blood clots in the menstrual period were identified as predictors of pregnancy.

**Conclusions/Implications for Practice:**

Health care providers should assist women planning to conceive in monitoring their menstrual cycles and devising strategies to optimize them, reduce dysmenorrhea and blood clots, and use ovulation test strips to enhance natural conceptions.

## Introduction

Postponing first-time pregnancy is a global trend that is particularly evident in the United States, Europe, and Taiwan. In Taiwan, for example, 30% of first-time mothers are over 35 years old (The American College of Obstetricians and Gynecologists, 2022; Department of Statistics, Ministry of the Interior, Taiwan, ROC, 2023; Eurostat, 2023). Furthermore, approximately one-sixth of women globally experience difficulties with fertility, with infertility rates of 10%–15% reported in developed nations (Eurostat, 2023; Hamilton et al., 2020; World Health Organization, 2024). Infertility is defined as an inability to conceive after 1 year of regular, unprotected intercourse (National Center for Chronic Disease Prevention and Health Promotion [U.S.], Division of Reproductive Health, 2021; World Health Organization, 2024). In 2018, around 13% of women of reproductive age in the United States sought fertility treatment. However, fewer than half with fertility difficulties are able to access fertility care (Hamilton et al., 2020; Peipert et al., 2022). In light of these issues, more inclusive, accessible, and affordable reproductive health services are needed (Delbaere et al., 2020).

Many women with fertility difficulties may be able to enhance their chances of conceiving naturally through lifestyle adjustments such as eating a balanced diet, performing regular exercise, and managing stress levels that support healthy menstruation (The American College of Obstetricians and Gynecologists, 2019; Delbaere et al., 2020; Eurostat, 2023; Hatcher & Namnoum, 2004; World Health Organization, 2024). Moreover, monitoring one’s menstrual cycle not only raises fertility awareness but also helps women identify their most fertile periods, maximizing their chances of conceiving. This is especially beneficial for those experiencing age-related fertility decline (Eurostat, 2023; Hatcher & Namnoum, 2004). However, many women suffering from infertility rely heavily on medical treatments and remain unaware of their menstrual cycle and its links with fertility (Diamond-Smith et al., 2020; National Center for Chronic Disease Prevention and Health Promotion [U.S.], Division of Reproductive Health, 2021; Passet-Wittig & Greil, 2021).

Menstruation is the monthly shedding of the uterine endometrial lining that coincides with periodic changes in the ovaries (Hatcher & Namnoum, 2004). The menstrual cycle is a precisely regulated process governed by the complex feedback mechanisms of the hypothalamic–pituitary–ovarian axis, which influences cycle length, bleeding, and ovulation timing (Cao et al., 2024). Five menstrual characteristics have been identified in the literature as being associated with time to pregnancy (TTP) and likelihood of conception. These include menstrual cycle length, number of days of bleeding, signs of ovulation, presence of dysmenorrhea, and presence of blood clots (Ried, 2015; Xiping et al., 2021). For example, women with normal cycle lengths (27–29 d) conceive around 4 months sooner than their peers with shorter or longer cycles (Bradley et al., 2019), and women from China with longer cycles were found to be less likely to conceive (Zhang et al., 2017). Other studies have found that women with a bleeding duration of 4–5 days tend to conceive sooner, while those with variable cycle lengths, heightened menstrual period pain, and more menstrual clots experience more difficulty conceiving (Bull et al., 2019; Cochrane et al., 2016). Dysmenorrhea and menstrual blood clots have been identified as potential indicators of uterine health problems that influence fertility (Cao et al., 2024).

Although a common belief is that ovulation occurs on Day 14 of the menstrual cycle, one study found that only 30% of women completed their entire fertile window within Days 10–17 of their cycle (The American College of Obstetricians and Gynecologists, 2019), while another found that at least 10% of women with regular cycles were within their fertile window between cycle Days 6 and 21 (Xiping et al., 2021). In another study on women aged 18–45 years from Sweden, the United Kingdom, and the United States, the mean ovulation period was 12.4 (*SD*=2.4) days, but it ranged from 8.0 days in women with 15–20-day cycles to 12.9 days in women with 36–50-day cycles (Bull et al., 2019). The findings of the above studies suggest that menstrual cycle length is not a sufficiently valid measure for predicting the ovulation period.

While some related studies have provided some insights into the association between menstrual characteristics and TTP, most have focused on a single menstrual attribute only. Thus, the multidimensional nature of menstrual characteristics and their relationships with conception have been largely overlooked. Furthermore, while variation in cycle length has generally been attributed to the timing of ovulation (Bull et al., 2019; Xiping et al., 2021), ovulation test strips have rarely been used to directly monitor ovulation timing in women preparing for pregnancy. Most research has been either retrospective (Bradley et al., 2019) or relied on data collected through anonymous online surveys (Bull et al., 2019), with the findings not yet supported by prospective study results.

As global fertility rates decline, promoting awareness and tracking of the menstrual cycle may be an effective approach to optimizing natural fertility potential (Cochrane et al., 2016). Therefore, the objectives of this study were to describe all five menstrual characteristics in detail and investigate their association with likelihood of conception within 6 months in women planning to become pregnant. The unique contributions of this study include its use of ovulation test strips, investigation of multidimensional measures of menstrual characteristics, and use of a prospective design to predict pregnancy.

## Methods

### Study Design and Participants

For this prospective panel study, 289 participants were recruited between August 2018 and July 2019. The inclusion criteria were ≥20 years of age, an intention to become pregnant within 1 year, being in a sexually active relationship, and not using birth control. Exclusion criteria were using hormonal therapy in the past 6 months, being unable to provide menstrual characteristics for the month before joining the study, having a physician-diagnosed genital disease, being infertile, and being unable to use basal thermometers and urine luteinizing hormone (LH) sampling ovulation test strips on a daily basis. Participants were recruited via online (Line and Facebook) advertisements.

In the initial face-to-face interview, demographics, parity, and age at menarche data were collected using a structured questionnaire. After completion, the participants were instructed on how to record their menstruation cycle and basal body temperature, as well as on how to use the ovulation test kit. Follow-up was then conducted monthly via telephone and email until data on at least the next three menstrual cycles had been obtained. If a participant reported a pregnancy, the date of her last menstrual period was documented. The participant’s pregnancy status was followed over the 6-month study period.

### Ethical Considerations

Before enrollment, a written informed consent form was signed by each participant. All of the participants were fully informed about the study and its voluntary nature and confidentiality guarantees. This study was approved by the institutional review board of National Yang-Ming University (YM106109E).

### Measures

A structured questionnaire was used to collect demographics (age and educational level), parity, and age at menarche data upon enrollment. Concurrently, the first author measured and recorded each participant’s height and weight to the nearest 0.1 cm and 0.1 kg, respectively. All of the measurements were taken while the participant was in a standing position and wearing light clothing and no shoes. Body mass index (BMI) was calculated and categorized, based on the guidelines of the World Health Organization, Regional Office for the Western Pacific (2000) for Asian populations, as follows: underweight, <18.5 kg/m^2^; normal weight, 18.5–22.9 kg/m^2^; overweight, 23.0–24.9 kg/m^2^; and obese, ≥ 25.0 kg/m^2^.

Upon enrollment, the participants recorded the characteristics of at least their next three menstrual cycles using the free Chinese-language smartphone app “Small Calendar.” Once the data recording was complete, it was sent to the first author via email for analysis. Menstrual cycle length was defined as the total number of days from the last day of menstrual bleeding to the day before the next onset of menstrual bleeding. “Days of bleeding” was defined as the number of days of menstrual bleeding (Bull et al., 2019; Zhang et al., 2017). The participants were required to track their ovulation every day upon waking using two methods: measuring their basal body temperature and using the ovulation test kit supplied at enrollment. In this study, signs of ovulation were defined as a drop in basal body temperature lasting 1–2 days accompanied by a positive ovulation test result. The accuracy of ovulation prediction based on self-measured basal body temperature is 74% (Guermandi et al., 2001), while ovulation test kits have proven sensitivities of 98%–100% for every-other-day testing (Guermandi et al., 2001; O’Connor et al., 2006). Based on previous studies, the participants were also asked to report the presence of dysmenorrhea and blood clots >2.4 cm in diameter (Passet-Wittig & Greil, 2021; United Kingdom National Health Service, 2024). Finally, the participants submitted a monthly pregnancy status update, and positive cases were confirmed by an obstetrician. The total TTP value was calculated as the interval between the first day of the last menstrual period at enrollment and the first day of the last menstrual period before conception.

### Sample Size and Statistical Power

A study conducted by Bradley et al. (2019) determined cycle-length variability to be associated with a 38% decrease in TTP within 12 months. Another preconception cohort study demonstrated that women over the age of 36 trying to conceive had a mean pregnancy rate of 46.3% within six cycles (Wesselink et al., 2017). Stata (version 15.0) statistical software was used to estimate the minimum required sample size, and Cox proportional hazards regression was used with an α value of .05, hazard ratio for pregnancy of 0.7, and pregnancy rate of 41%. This yielded a minimum sample size of 255 needed to achieve a statistical power of 0.80. At the end of the study period, 129 participants (44.6%) had been confirmed to be pregnant; with this sample size, the statistical power of the study exceeded 80%.

### Data Analysis

Data analyses were performed using IBM SPSS Statistics 22.0 (IBM Inc., Armonk, NY, USA) and SAS version 9.4 (SAS Institute, Cary, NC, USA). The Group-Based Trajectory Modeling (GBTM) method was employed to group the participants by menstrual pattern, with each participant assigned to one group only for each menstrual characteristic. The menstrual characteristics were identified using the Censored Normal (CNORM) model with GBTM in SAS, which classifies heterogeneous populations into more homogeneous clusters or classes. GBTM is a flexible semi-parametric mixture model used to discover latent patterns of change over time and estimate the group assignment probabilities. The optimum numbers of groups and dimensions were determined based on the lowest Bayesian Information Criterion values, the average of maximum probabilities >70%, and the proportion of estimated trajectory classes (the smallest group includes at least 5% of individuals). Quadratic functions were used to estimate menstrual characteristic trajectories (Brame et al., 2006; Jones et al., 2001).

Descriptive analyses were performed by calculating frequencies, percentages, means, and standard deviations; menstrual characteristics were examined based on pregnancy rates within the 6-month study period using χ^2^ tests; and the Cox proportional hazards model was used to assess the fecundability ratios (FRs) and 95% CIs using incident pregnancy as the dependent variable. To achieve a more parsimonious model, as recommended by Bentler and Mooijaart (1989), only variables with a significant correlation (*p*<.05) were retained in the model. Finally, the Kaplan–Meier method was used to compute the TTP during the observation period. All of the conducted tests were two-sided, with *p*<.05 considered statistically significant.

## Results

The age range of the 289 participants was 20–42 years (mean: 32.0±5.0). Most had at least a college-level education, the average pre-pregnancy BMI was 22.5±3.8 kg/m^2^ (range: 15.8–38.3), and the average age at menarche was 13.0±1.6 years (range: 9–18; Table 1).

**Table 1:** Participant Characteristics (*N*=289)

Variable	*n* (%)
Age (years; *M* and *SD*)	32.0 (5.0)
<30	102 (35.3)
30–35	114 (39.4)
≥36	73 (25.3)
Educational level	
High school or lower	14 (4.8)
College or above	275 (95.2)
Parity	
Primigravida	179 (61.9)
Multigravida	110 (38.1)
BMI category (kg/m^2^, *M* and *SD*)	22.5 (3.8)
Underweight (<18.5)	31 (10.8)
Normal (18.5–22.9)	146 (50.5)
Overweight (23.0–24.9)	48 (16.6)
Obese (≥25.0)	64 (22.1)
Age at menarche (*M* and *SD*)	13.0 (1.6)
Pregnant within 6 months	129 (44.6)
Menstrual cycle length (days)	
≤26	40 (13.8)
27–32	174 (60.2)
33–47	43 (14.9)
≥48	32 (11.1)
Days of bleeding (days)	
≤4	33 (11.5)
5–6	205 (70.9)
≥7	51 (17.6)
Ovulation signs	
No presence	26 (9.0)
Presence 13–16​​​​​​ days	226 (78.2)
Presence ≤12 days or ≥17 days	37 (12.8)
Dysmenorrhea and clots	
None	156 (54.0)
Clots only	19 (6.6)
Dysmenorrhea only	57 (19.7)
Both	57 (19.7)

During the study period, 1,321 menstrual cycles were recorded. Using the GBTM method, the participants were assigned to four, three, and three groups, respectively, based on menstrual cycle length, number of days of bleeding, and signs of ovulation (Table 1). The most common cycle length was 27–32 days (60.2% of participants), the most common number of days of bleeding was 5–6 (70.9%), and the most common range of cycle days on which ovulation signs were present was 13–16 (78.2%). As the presence of dysmenorrhea and blood clots was significantly correlated (*r*=.44, *p*<.001), to avoid collinearity, these two variables cannot be entered as independent variables in the model. Examining both variables together showed that 54% of the participants did not experience either symptom, 19.7% experienced both, 19.7% experienced dysmenorrhea only, and 6.6% experienced clots only (*n*=19). Given the small size of the clots-only group, the four groups were combined into two groups, respectively indicating the presence or absence of either dysmenorrhea or blood clots in further analysis. The 129 participants (44.6%) confirmed to be pregnant during the 6-month study period had a mean TTP of 81.9 (*SD*=38.1) days (Table 1).

The crude and adjusted results for factors related to becoming pregnant are presented in Tables 2 and 3. Being 30–35 years old, having a cycle length of 27–32 days, experiencing menstrual bleeding for 5–6 days, having signs of ovulation on Days 13–16, and not having dysmenorrhea and clots were each found to be associated with a higher likelihood of becoming pregnant (all *p*<.001; Table 2). Those variables retained their significance in the multivariate models for incident pregnancy (Table 3).

**Table 2:** Comparison of Menstrual Characteristics of Participants Who Did and Did Not Become Pregnant (*N*=289)

Variable	Pregnant Within 6 Months		χ^2^/*t*	*p*
	Yes (*n*=129)	No (*n*=160)		
	*n* (%)	*n* (%)		
Age (years)			9.7	.008^*^
<30	34 (33.3)	68 (66.7)		
30–35	62 (54.4)	52 (45.6)		
≥36	33 (45.2)	40 (54.8)		
Educational level			2.3	.129
High school or lower	9 (64.3)	5 (35.7)		
College or above	120 (43.6)	155 (56.4)		
Parity			0.2	.643
Primigravida	78 (43.6)	101 (56.4)		
Multigravida	51 (46.4)	59 (53.6)		
Age at menarche (years, *M* and *SD*)	12.9±1.6	13.1±1.7	0.6	.521
Asian pre-pregnancy BMI category (kg/m^2^)			0.3	.962
Underweight (<18.5)	14 (45.2)	17 (54.8)		
Normal (18.5–22.9)	63 (43.2)	83 (56.8)		
Overweight (23.0–24.9)	22 (45.8)	26 (54.2)		
Obese (≥25.0)	30 (46.9)	34 (53.1)		
Menstrual cycle length (days)			36.4	<.001^**^
≤26	6 (15.0)	34 (85.0)		
27–32	102 (58.6)	72 (41.4)		
33–47	12 (27.9)	31 (72.1)		
≥48	9 (28.1)	23 (71.9)		
Days of bleeding (days)			40.5	<.001^**^
≤4	13 (39.4)	20 (60.6)		
5–6	113 (55.1)	92 (44.9)		
≥7	3 (5.9)	48 (94.1)		
Ovulation signs			44.1	<.001^**^
None	3 (11.5)	23 (88.5)		
Present on Days 13–16	124 (54.9)	102 (45.1)		
Present before or after Days 13–16	2 (5.4)	35 (94.6)		
Dysmenorrhea or clots			25.7	<.001^**^
Absent	91 (58.3)	65 (41.7)		
Present	38 (28.6)	95 (71.4)		

^*^ *p*<.05. **p<.001.

**Table 3:** Cox Proportional Hazards Regression of the Associations Among Age, Menstrual Characteristics, and Conception Within 6 Months (*N*=289)

Variable	Crude FRs	95% CI	*p*	Adjusted FRs	95% CI	*p*
Age (years)						
<30 ^a^	1.00			1.00		
30–35	1.86	[1.23, 2.83]	.004^*^	1.85	[1.22, 2.82]	.004^*^
≥36	1.48	[0.92, 2.40]	.107	1.40	[0.86, 2.28]	.180
Menstrual cycle length (days)						
≤26	0.20	[0.09, 0.44]	<.001^**^	0.19	[0.08, 0.43]	<.001^**^
27–32 ^a^	1.00			1.00		
33–47	0.38	[0.21, 0.70]	.002^*^	0.36	[0.20, 0.66]	.001^*^
≥48	0.40	[0.20, 0.79]	.008^*^	0.43	[0.22, 0.86]	.016^*^
Days of bleeding (days)						
≤4	0.62	[0.35, 1.09]	.098	0.60	[0.33, 1.07]	.082
5–6 ^a^	1.00			1.00		
≥7	0.08	[0.02, 0.24]	<.001^**^	0.07	[0.02, 0.23]	<.001
Ovulation signs						
None	0.15	[0.05, 0.48]	.001^*^	0.16	[0.05, 0.50]	.002^*^
Present on Days 13–16 ^a^	1.00			1.00		
Present before or after Days 13–16	0.07	[0.02, 0.28]	<.001^**^	0.07	[0.02, 0.27]	<.001^**^
Dysmenorrhea or clots						
Absent	1.00			1.00		
Present	0.36	[0.25, 0.52]	<.001^**^	0.35	[0.24, 0.52]	<.001^**^

*Note.* The model was adjusted for educational level (high school or lower, college or above), parity, age at menarche, and Asian pre-pregnancy BMI category. FRs = fecundability ratios; CI = confidence interval.

^a^ Reference category.

^*^ *p* <.05. **p <.001.

**Figure 1:**
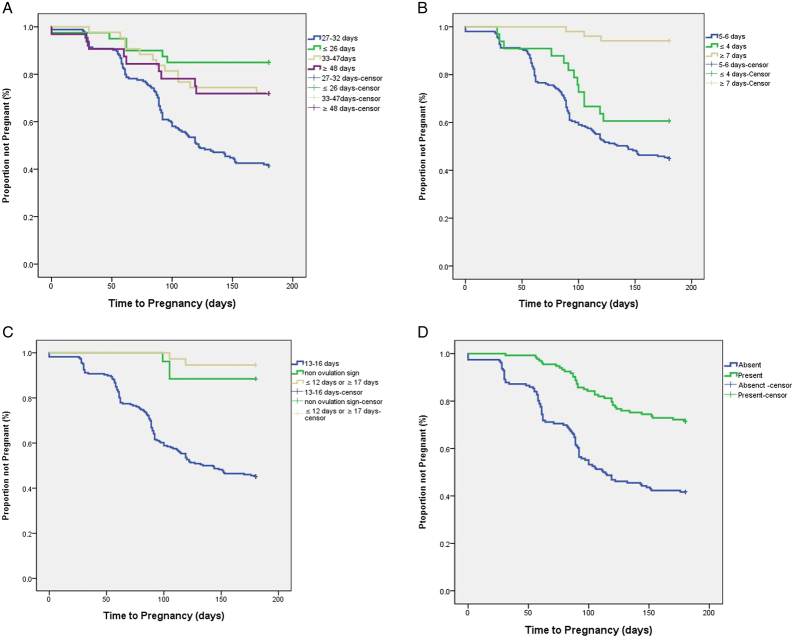
Kaplan–Meier Survival Curve Showing the Relationship Between Time to Pregnancy and (A) Menstrual Cycle Length, (B) Number of Days of Bleeding, (C) Ovulation Signs, and (D) Dysmenorrhea and Blood Clots

The results of the Kaplan–Meier analysis indicated that the menstrual characteristic groups with the highest pregnancy rates and FRs also had a significantly shorter TTP (Table 4). Women with a menstrual cycle length of 27–32 days, 5–6 days of bleeding, ovulation signs on Days 13–16, and without dysmenorrhea or clots all had a shorter mean TTP (Figure 1).

**Table 4:** Kaplan–Meier Analysis of Relationships Between Menstrual Characteristics and Time to Pregnancy (*N*=289)

Characteristic	Time to Pregnancy (days)				Log Rank
	Estimate	*SE*	95% CI	Median (*SD*)	*p*
Menstrual cycle length (days)				122.0 (13.6)	<.001**
≤26	162.0	7.0	[148.2, 175.8]		
27–32	123.7	4.2	[115.4, 132.0]		
33–47	154.0	6.9	[140.4, 167.6]		
≥48	148.2	9.7	[129.1, 167.2]		
Days of bleeding (days)				143.0 (22.2)	<.001**
≤4	142.2	8.9	[124.8, 159.5]		
5–6	125.5	4.0	[117.6, 133.3]		
≥7	175.6	2.5	[170.6, 180.0]		
Ovulation signs				134.0 (0.0)	<.001**
None	171.1	4.8	[161.7, 180.0]		
Present on Days 13–16	125.6	3.8	[118.2, 133.1]		
Present before or after Days 13–16	176.3	2.5	[171.3, 180.0]		
Dysmenorrhea or clots				112.0 (18.0)	<.001**
Absent	118.1	4.8	[108.8, 127.5]		
Present	157.5	3.5	[150.6, 164.3]		

*Note.* Time to pregnancy in days was calculated as the interval between the first day of the last menstrual period before enrollment and the first day of the last menstrual period before conception.

** *p*<.001.

## Discussion

### Menstrual Characteristics and TTP

Our findings that a cycle length of 27–32 days and 5–6 days of bleeding per cycle relate to a higher chance of becoming pregnant and a shorter TTP are in line with previous studies (Bentler & Mooijaart, 1989; Cochrane et al., 2016; Wesselink et al., 2017; Zhang et al., 2017), although our categories differed from those used previously. For example, one study reported that women in China are most fertile when their cycles last 27–29 days and they bleed for 4–5 days (Zhang et al., 2017), while another reported that bleeding for >7 days is associated with lower fertility (Small et al., 2006). These differences relate primarily to the timing of ovulation. In this study, to accurately identify the timing of ovulation and improve understanding of menstrual cycle variability, menstrual patterns were tracked closely and prospectively. The use of a prospective design and the GBTM method allowed the objective classification of menstrual patterns and provided new insights into their relationship with TTP. This approach is more objective than the currently used approaches, providing a useful foundation for future research. Despite these small differences in categories used, the findings of this study support prior research showing that cycle length and number of bleeding days relate significantly to conception. Understanding these relationships can empower women to make informed decisions about their reproductive health and pregnancy planning (Bradley et al., 2019; Bull et al., 2019). This study is novel in using basal body temperature and LH test strips to help women better identify their fertile windows. Given this understanding, women can maximize their chances of conception by tracking their cycles carefully and using basal body temperature and LH test strips to identify their own fertile windows. These measures are easy to implement and thus have a high likelihood of acceptance. Future efforts should focus on increasing preconception counseling, which should include guiding women to monitor their menstrual cycles closely (Small et al., 2006; Xiping et al., 2021).

Ovulation signs on Days 13–16 and a cycle length of 27–32 days were identified in this study as significantly associated with a shorter TTP, which corresponds with data from the United Kingdom (cycle length of 23–35 days and ovulation signs on Days 10–15 associated with shorter TTP; Soumpasis et al., 2020) and an international study (shorter TTP associated with a cycle length of 25–30 days and ovulation signs on Day 12.4±2.4 days or a cycle length of 25–35 days and ovulation signs on Days 12.4±2.2 to 12.9±2.3; Bull et al., 2019). In previous studies, the day of ovulation was identified using blood tests and ultrasound (Chinta et al., 2020; Tsuchida et al., 2022). However, basal body temperature and LH test strips are more convenient for use at home (Bull et al., 2019) and appear to be highly accurate (Lei et al., 2023; Yu et al., 2022). Nurses may teach women planning to become pregnant to use LH test strips to increase their chance of pregnancy.

### Dysmenorrhea, Blood Clots, and Fertility

Nearly 50% of participants who experienced dysmenorrhea and/or blood clots exhibited significantly lower rates of fertility than their peers who did not. Following the UK’s National Health Service (2024), we consider menstrual blood clots larger than 2.4 cm in diameter as indicative of potential uterine health problems and poor lifestyle habits, with previous research showing these symptoms may indicate reproductive system diseases and a longer TTP duration (Ried, 2015; Xiping et al., 2021). No significant association has yet been found between the degree of dysmenorrhea or menstrual blood clot size and fertility. The results of this study indicate that more attention should be given to these relationships in future research. Visible or frequent clots may indicate conditions such as uterine polyps or fibroids, endometriosis, adenomyosis, and hormonal imbalances (Xiping et al., 2021), and nurses may advise women with those conditions to seek related medical care. Encouraging women to make lifestyle adjustments such as managing their weight, taking appropriate exercise, and getting adequate sleep can help to reduce these conditions and improve reproductive health (Eurostat, 2023; United Kingdom National Health Service, 2024; Xiping et al., 2021).

### Multidimensional Menstrual Characteristics, TTP, and Reproductive Health

The multidimensional characteristics of the menstrual cycle serve as a series of critical indicators for reproductive health (The American College of Obstetricians and Gynecologists, 2019; Cao et al., 2024). Reproductive hormones influence the stability of cycle length, ovulation, and menstrual blood volume, while pain and blood clots reflect uterine circulatory health, thereby affecting implantation rates (Lei et al., 2023; Ried, 2015). Moreover, Bull et al. (2019) suggested that menstrual monitoring not only enhances conception rates but also improves reproductive health by allowing individuals to observe menstrual changes. Therefore, reproductive health assessments should include a comprehensive assessment of menstruation. Nurses may use the findings reported in this study to help women understand their reproductive health and increase their chance of natural conception.

In this study, the participants who became pregnant had a mean TTP of 81.9 (*SD*=38.1) days. Most studies examining TTP have been retrospective and either involved participant self-reports or a calculation based on when contraception use was stopped (Bull et al., 2019; Wesselink et al., 2017; Zhang et al., 2017), both of which introduce the risk of recall bias. In this prospective study, participants were confirmed not to be pregnant at baseline, and TTP was calculated based on the first day of the baseline cycle (Wesselink et al., 2017; Zhang et al., 2017), suggesting a more accurate and reliable approach.

The participants in this study were older and had a higher educational level than the general population, which is not surprising, as they were all women planning to become pregnant. The finding that participants aged 30–35 years were more likely to become pregnant than those under 30 may be because they adopted relatively more healthy behaviors (McDougall et al., 2021). However, further studies are needed to examine this speculation. The pre-pregnancy BMI of the participants was close to the average pre-pregnancy BMI (23.0±0.3 kg/m^2^) of women in a similar age range (19–44 years) in Taiwan (Health Promotion Administration, Ministry of Health and Welfare, Taiwan, ROC, 2018). The relationship between pre-pregnancy BMI and fertility reported in previous research (Fang et al., 2020) was not found in this study. This may be because approximately half of the participants had a normal pre-pregnancy BMI. Small observational studies such as this one often lack sufficient statistical power to detect the effects of BMI on TTP. Future research involving larger sample sizes may better elucidate this relationship in Asian populations.

### Study Limitations

First, the participants were asked to self-report their menstrual characteristics for at least three menstrual cycles, potentially introducing coverage error or selection bias. Second, the use of ovulation test strips and thermometers may have affected fertility awareness and thus reduced the comparability of our results with previous retrospective data–based findings. However, the research design used in this study was expected to accurately record changes in all aspects of the participants’ menstrual characteristics, with these data providing an empirical basis and reference for future research. Finally, the focus of this study was primarily on assessing the impact of the five menstrual characteristics on fertility, and it did not collect information on other relevant factors, such as changes in desire to become pregnant or frequency of sexual activity during the study period. Collecting these and other data in future research will help provide a more comprehensive understanding of the influence of menstrual characteristics on fertility in women of reproductive age.

### Conclusions

The “pro-pregnancy” menstruation characteristics identified in this study include having a menstrual cycle length of 27–32 days, menstrual bleeding lasting 5–6 days, signs of ovulation on Days 13–16, and the absence of dysmenorrhea and clots during menstruation. During preconception clinical visits, health care providers should develop strategies to help women who desire to conceive experience healthier menstrual cycles, reduce their dysmenorrhea and blood clots, and monitor their signs of ovulation based on basal body temperature and ovulation test kits, thus increasing their chances of natural conception. Future randomized controlled trials are needed to confirm whether healthier menstrual characteristics significantly increase the chances of becoming pregnant.

## References

[R1] The American College of Obstetricians and Gynecologists. (2019). Prepregnancy counseling: Committee opinion. Fertility and Sterility, 111(1), 32–42. 10.1016/j.fertnstert.2018.12.003 30611411

[R2] The American College of Obstetricians and Gynecologists. (2022). *Having a baby after age 35: How aging affects fertility and pregnancy* (ACOG Publication No. FAQ060). The American College of Obstetricians and Gynecologists. Retrieved from https://www.acog.org/womens-health/faqs/having-a-baby-after-age-35-how-aging-affects-fertility-and-pregnancy

[R3] Bentler P. M. Mooijaart A. (1989). Choice of structural model via parsimony: A rationale based on precision. Psychological Bulletin, 106(2), 315–317. 10.1037/0033-2909.106.2.315 2678203

[R4] Bradley D. Landau E. Jesani N. Mowry B. Chui K. Baron A. Wolfberg A. (2019). Time to conception and the menstrual cycle: An observational study of fertility app users who conceived. Human Fertility, 24(4), 267–275. 10.1080/14647273.2019.1613680 31094573

[R5] Brame R. Nagin D. S. Wasserman L. (2006). Exploring some analytical characteristics of finite mixture models. Journal of Quantitative Criminology, 22, 31–59. 10.1007/s10940-005-9001-8

[R6] Bull J. R. Rowland S. P. Scherwitzl E. B. Scherwitzl R. Danielsson K. G. Harper J. (2019). Real-world menstrual cycle characteristics of more than 600,000 menstrual cycles. NPJ Digital Medicine, 2(1), 83–91. 10.1038/s41746-019-0152-7 31482137 PMC6710244

[R7] Cao Y. Zhao X. Dou Z. Gong Z. Wang B. Xia T. (2024). The correlation between menstrual characteristics and fertility in women of reproductive age: A systematic review and meta-analysis. Fertility and Sterility, 122(5), 918–927. 10.1016/j.fertnstert.2024.06.016 38936536

[R8] Chinta P. Rebekah G. Kunjummen A. T. Kamath M. S. (2020). Revisiting the role of serum progesterone as a test of ovulation in eumenorrheic subfertile women: A prospective diagnostic accuracy study. Fertility and Sterility, 114(6), 1315–1321. 10.1016/j.fertnstert.2020.06.030 32943223

[R9] Cochrane S. Smith C. A. Possamai-Inesedy A. Bensoussan A. (2016). Prior to conception: The role of an acupuncture protocol in improving women’s reproductive functioning assessed by a pilot pragmatic randomised controlled trial. Evidence-Based Complementary and Alternative Medicine, 2016(1), Article 3587569. 10.1155/2016/3587569 PMC4868913 27242910

[R10] Delbaere I. Verbiest S. Tydén T. (2020). Knowledge about the impact of age on fertility: A brief review. Upsala Journal of Medical Sciences, 125(2), 167–174. 10.1080/03009734.2019.1707913 31964217 PMC7721003

[R11] Department of Statistics, Ministry of the Interior, Taiwan, ROC. (2023). *Population by marital status.* Retrieved from https://ws.moi.gov.tw/001/Upload/400/relfile/0/4405/48349492-6f8c-453b-a9d1-4a8f0593b979/year/year.html (Original work published in Chinese)

[R12] Diamond-Smith N. Onyango G. O. Wawire S. Ayodo G. (2020). Knowledge of menstruation and fertility among adults in rural Western Kenya: Gaps and opportunities for support. PLOS One, 15(3), Article e0229871. 10.1371/journal.pone.0229871 PMC7053729 32126117

[R13] Eurostat. (2023). Fertility statistics. European Commission. https//ec.europa.eu/eurostat/statistics-explained/index.php/Fertility_statistics

[R14] Fang Y. Liu J. Mao Y. He Y. Li M. Yang L. Zhou W. (2020). Pre-pregnancy body mass index and time to pregnancy among couples pregnant within a year: A China cohort study. PLOS One, 15(4), Article e0231751. 10.1371/journal.pone.0231751 PMC7179844 32324768

[R15] Guermandi E. Vegetti W. Bianchi M. M. Uglietti A. Ragni G. Crosignani P. (2001). Reliability of ovulation tests in infertile women. Obstetrics & Gynecology, 97(1), 92–96. 10.1016/s0029-7844(00)01083-8 11152915

[R16] Hamilton B. E. Martin J. A. Osterman M. J. K. Division of Vital Statistics, National Center for Health Statistics. (2020). Births: Provisional data for 2019. Vital Statistics Rapid Release, (8), 1–10. Retrieved from https://www.cdc.gov/nchs/data/vsrr/vsrr-8-508.pdf 33814033

[R17] Hatcher R. A. Namnoum A. B. (2004). The menstrual cycle. Contraceptive Technology, 18, 63–72.

[R18] Health Promotion Administration, Ministry of Health and Welfare, Taiwan ROC. (2018). *19-44 years women body mass index.* Retrieved from https://www.gender.ey.gov.tw/gecdb/Stat_Statistics_DetailData.aspx?sn=%24mQvpHYEayTTt8pmhMjRvA%40%40 (Original work published in Chinese)

[R19] Jones B. L. Nagin D. S. Roeder K. (2001). A SAS procedure based on mixture models for estimating developmental trajectories. Sociological Methods & Research, 29(3), 374–393. 10.1177/0049124101029003005

[R20] Lei Z. Feng Y. Junzhe Z. (2023). LH Ovulation Rapid Test Cassette (urine) plays an important role in preparing pregnant. Health Science Journal, 17(1), Article 991. 10.36648/1791-809X.17.1.991

[R21] McDougall B. Kavanagh K. Stephenson J. Poston L. Flynn A. C. White S. L. (2021). Health behaviours in 131,182 UK women planning pregnancy. BMC Pregnancy and Childbirth, 21, Article No. 530. 10.1186/s12884-021-04007-w PMC8317296 34315424

[R22] National Center for Chronic Disease Prevention and Health Promotion (U.S.), Division of Reproductive Health. (2021). *Assisted reproductive technology—Fertility clinic success rates report* (CDC Publication No. 154438). Retrieved from https://stacks.cdc.gov/view/cdc/107280/cdc_107280_DS1.pdf

[R23] O’Connor K. Brindle E. Miller R. Shofer J. Ferrell R. Klein N. Wood J. (2006). Ovulation detection methods for urinary hormones: Precision, daily and intermittent sampling and a combined hierarchical method. Human Reproduction, 21(6), 1442–1452. 10.1093/humrep/dei497 16439502

[R24] Passet-Wittig J. Greil A. L. (2021). Factors associated with medical help-seeking for infertility in developed countries: A narrative review of recent literature. Social Science & Medicine, 277, Article 113782. 10.1016/j.socscimed.2021.113782 33895708

[R25] Peipert B. J. Chung E. H. Harris B. S. Warren C. M. Jain T. (2022). Impact of comprehensive state insurance mandates on in vitro fertilization utilization, embryo transfer practices, and outcomes in the United States. American Journal of Obstetrics and Gynecology, 227(1), 64 e1–e8. 10.1016/j.ajog.2022.03.003 35283088

[R26] Ried K. (2015). Chinese herbal medicine for female infertility: An updated meta-analysis. Complementary Therapies in Medicine, 23(1), 116–128. 10.1016/j.ctim.2014.12.004 25637159

[R27] Small C. M. Manatunga A. K. Klein M. Feigelson H. S. Dominguez C. E. McChesney R. Marcus M. (2006). Menstrual cycle characteristics: Associations with fertility and spontaneous abortion. Epidemiology, 17(1), 52–60. 10.1097/01.ede.0000190540.95748.e6 16357595

[R28] Soumpasis I. Grace B. Johnson S. (2020). Real-life insights on menstrual cycles and ovulation using big data. Human Reproduction Open, 2020(2), Article hoaa011. 10.1093/hropen/hoaa011 PMC7164578 32328534

[R29] Tsuchida M. Komura N. Yoshihara T. Kawasaki Y. Sakurai D. Suzuki H. (2022). Ultrasonographic observation in combination with progesterone monitoring for detection of ovulation in labrador retrievers. Reproduction in Domestic Animals, 57(2), 149–156. 10.1111/rda.14035 34724259

[R30] United Kingdom National Health Service. (2024). Heavy period. United Kingdom National Health Service. Retrieved from https://www.nhs.uk/conditions/heavy-periods/

[R31] Wesselink A. K. Rothman K. J. Hatch E. E. Mikkelsen E. M. Sørensen H. T. Wise L. A. (2017). Age and fecundability in a North American preconception cohort study. American Journal of Obstetrics and Gynecology, 217(6), 667e1–667e8. 10.1016/j.ajog.2017.09.002 PMC5712257 28917614

[R32] World Health Organization, Regional Office for the Western Pacific. (2000). The Asia-Pacific perspective: Redefining obesity and its treatment. Health Communications Australia. Retrieved from https://iris.who.int/handle/10665/206936World

[R33] World Health Organization. (2024). Infertility. World Health Organization. Retrieved from https://www.who.int/news-room/fact-sheets/detail/infertility

[R34] Xiping L. Xiaqiu W. Lirong B. Jin P. Hui K. K. (2021). Menstrual cycle characteristics as an indicator of fertility outcomes: Evidence from prospective birth cohort study in China. Journal of Traditional Chinese Medicine, 42(2), 272–279. 10.19852/j.cnki.jtcm.2022.02.010 PMC9924698 35473349

[R35] Yu J.-L. Su Y.-F. Zhang C. Jin L. Lin X.-H. Chen L.-T. Huang H.-F. Wu Y.-T. (2022). Tracking of menstrual cycles and prediction of the fertile window via measurements of basal body temperature and heart rate as well as machine-learning algorithms. Reproductive Biology and Endocrinology, 20(1), Article No. 118. 10.1186/s12958-022-00993-4 PMC9375297 35964035

[R36] Zhang Q. Wang Y. Zhang Y. Zhang H. Yang Y. He Y. Xu J. H. Zhao J. Peng Z. Q. Ma X. (2017). The influence of age at menarche, menstrual cycle length and bleeding duration on time to pregnancy: A large prospective cohort study among rural Chinese women. BJOG: An International Journal of Obstetrics & Gynaecology, 124(11), 1654–1662. 10.1111/1471-0528.14469 28128508

